# Lack of anabolic response to skeletal loading in mice with targeted disruption of the pleiotrophin gene

**DOI:** 10.1186/1756-0500-1-124

**Published:** 2008-12-01

**Authors:** Chandrasekhar Kesavan, Subburaman Mohan

**Affiliations:** 1 Musculoskeletal Disease Center, VA Loma Linda Healthcare System, Loma Linda, CA 92357, USA; 2 Department of Medicine, Loma Linda University, Loma Linda, CA 92357, USA

## Abstract

**Background:**

In a previous study we showed, using the whole genome microarray approach, that pleiotrophin (PTN) expression was increased by 4-fold in response to mechanical loading (ML) in a good responder C57BL/6J (B6) mice. To address PTN role in mediating ML effects on bone formation, we first evaluated time course effects of ML on expression levels of PTN gene using real time RT-PCR in 10 week female B6 mice. A 9 N load was applied using a four-point bending device at 2 Hz frequency for 36 cycles, once per day for 2, 4 and 12 days on the right tibia and the left tibia was used as internal control.

**Findings:**

Four-point bending caused an acute increase in PTN expression (2-fold) within 2 days of loading and further increased (3–6 fold) with continued loading. This increase was also seen in 16 and 36-week old mice. Based on these findings, we next used PTN knockout (KO) mice to evaluate the cause and effect relationship. Quantitative analysis showed that two weeks of ML induced changes in vBMD and bone size in the PTN KO mice (8% and 6% vs. non-loaded bones) were not significantly different from control mice (11% and 8% in vBMD and bone size vs. non-loaded bones).

**Conclusion:**

Our results imply that PTN is not a key upstream mediator of the anabolic effects of ML on the skeleton.

## Findings

### Background

Mechanical loading is now recognized as an important stimulator of bone formation. Numerous studies in animal and humans, using various loading models have demonstrated that loading increases bone mass while unloading decreases bone mass [1-6]. To date, reports have shown that several growth factors and signaling pathways are known to be activated by ML [7-11]. However, the relative contribution of each of these pathways to ML induced bone formation is not known. We previously, using genome-wide microarray approach have reported that mechanical loading by four-point bending caused a 4-fold increase in Heparin binding growth factor, otherwise known as PTN, in a good responder B6 mouse [7]. PTN, a 36 amino acid bone growth factor rich in lysine and cysteine residues, is also known as Osteoblast Specific Factor 1. PTN is involved in diverse functions, which includes: cell recruitment, cell attachment and proliferation, differentiation, angiogensis, and neurogenesis [12-14]. *In vitro *studies have demonstrated that PTN has the ability to promote adhesion, migration, expansion and differentiation of human osteoprogenitor and MC3T3-E1 cells [15-17]. *In vivo *studies using transgenic approach have shown that ovariectomy induced bone loss, due to estrogen deficiency, were protected by an increase in the expression of the PTN gene [18]. Another transgenic study, with overexpression of the human PTN gene showed an increase in cortical thickness, bone volume and cancellous bone volume [15]. In addition, immunocytochemistry studies has provided visual evidence for PTN at the site of new bone formation [15,16]. Based on the above findings and our data that PTN expression is increased in response to ML, we hypothesize that PTN play a role in mediating anabolic effects of ML on bone formation. To test this hypothesis, we performed ML using four-point bending device on mice with disruption of PTN gene and control mice with intact PTN gene.

## Methods

### Mice

Female C57BL/6J (B6) mice were purchased from Jackson laboratory (Bar Harbor, ME). PTN gene knock out (KO) mice (PTN-129 in B6 background) were generated by Dr. Thomas F. Vogt and the breeding pairs were kindly provided by Princeton University, NJ, USA, for our studies. PTN KO mice were crossed with wild type B6 mice to generate the heterozygotes. These were crossed with each other to generate 25% homozygous PTN KO mice, 50% heterozygous and 25% littermate wild type mice. The body weight of PTN KO and control mice used for this study are 18.20 ± 0.95 g and 19.0 ± 1.39 g, respectively. The differences in body weight were not statistically significant (p = 0.20). All mice were housed under the standard conditions of 14-hour light and 10-hour darkness, and had free access to food and water. The experimental protocols were in compliance with animal welfare regulations and approved by local IACUC.

### Genotyping

At 3-weeks (wks) of age, DNA was extracted from tail of female mice, using a PUREGENE DNA purification kit (Gentra System, Inc., Minneapolis, MN) according to the manufacturer's protocol. Polymerase chain reaction (PCR) was performed to identify PTN KO mice from wild type or heterozygous mice. Primers specific for neomycin gene (forward 5' CTT GCT CCT GCC GAG AAA GTA T 3' and reverse 5' AGC AAT ATC ACG GGT AGC CAA C 3' with a PCR product of 369 bp). Primers specific for PTN gene (forward 5' TCT GAC TGT GGQA GAA TGG CAG T 3' and reverse 5' CTT CTT CCA GTT GCA AGG GAT C 3' with a PCR product of 147 bp) were used for genotyping. The following conditions were used to perform the PCR reaction: 95°C for 2 minutes; 35 cycles at 95°C for 40 sec, 57°C for 40 sec, 72°C for 40 sec; 70°C for 40 sec. The PCR products were run on a 1.5% agarose gel and the image taken with a ChemiImager 4400 (Alpha Innotech Corp., San Leandro, CA).

### *In vivo *loading model/regimen

ML was performed using a four-point bending device [Instron, Canton, MA], as previously reported [1]. The mice were loaded using a 9.0 ± 0.2 Newton (N) force at a frequency of 2 Hz for 36 cycles, once a day under inhalable anesthesia (5% Isoflurane and 95% oxygen). The right tibia was used for loading and the left tibia as internal non-loaded control.

For the time course study, the loading was performed at 2-, 4- and 12-days on 10-week female B6 mice. After 24 hours of the last loading, mice were euthanized and tibiae were collected for RNA extraction.

For varying age groups of female B6 mice, female PTN KO and control mice, the loading was performed for 12 days. After 48 hours of the last loading, following *in vivo *bone measurement, mice were euthanized; tibiae (loaded and non-loaded) were collected and stored at -80°C for further experiments.

### Peripheral quantitative computed tomography (pQCT) measurements

To measure four-point bending induced changes in the bone parameters in loaded and non-loaded tibiae, we used pQCT (Stratec XCT 960 M, Norland Medical System, Ft. Atkinson, WI) as described previously [1].

### RNA extraction

RNA was extracted from the loaded and non-loaded bones using qiagen lipid extraction kit [Qiagen, Valencia, CA], as previously described [1]. Quality and quantity of RNA were analyzed using the 2100 Bio-analyzer (Agilent, Palo Alto, CA, USA) and Nano-drop (Wilmington, DE).

### Reverse Transcriptase – Real time PCR

Using 200 ng purified total RNA, first strand cDNA was synthesized by iScript cDNA synthesis kit (BIO-RAD, CA, USA), according to the manufacturer's protocol. Quantitative real time PCR was performed, as previously described, in order to analyze the expression levels of PTN and PPIA ((peptidylprolyl isomerase A), an endogenous control) [1]. The data were analyzed using SDS software, version 2.0, and the results were exported to Microsoft Excel for further analysis. Data normalization was accomplished using the endogenous control (PPIA) to correct for variation in the RNA quality among samples. The normalized Ct values were subjected to a 2^-ΔΔ^Ct formula to calculate the fold change between the loaded and non -loaded groups. The formula and its derivations were obtained from the instrument user guide.

### Statistical Analysis

Values are given as mean ± SD. ANOVA (Bonferroni's post-hoc test) and standard t-test were used to compare the difference between load and non-loaded bones at various time-points, ages and strains using the fold change and percentage data. We used Statistica software (StatSoft, Inc version 7.1, 2005) to perform the analysis and the results were considered significant at p < 0.05.

## Results and discussion

**Figure 1:**
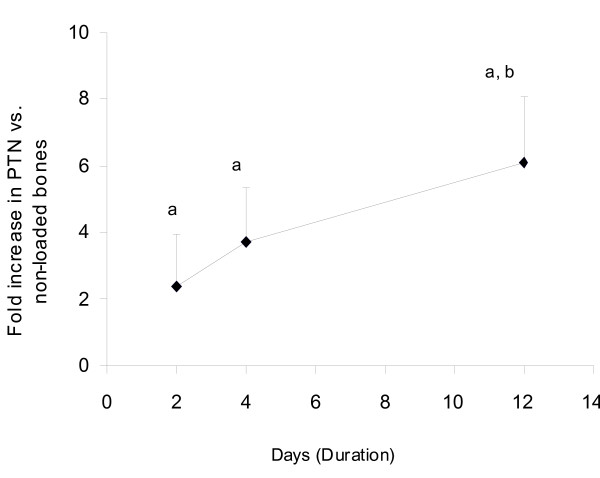
**Expression levels of PTN gene as a function of duration of loading**. The y-axis represents fold increase in the PTN gene in response to four-point bending on tibia and the x-axis represents duration of loading on 10-week female B6 mice. Values are mentioned as mean ± SD, ^a^p < 0.01 vs. non-loaded tibiae, ^b^p < 0.05 vs. 2-days (Post Hoc test, ANOVA), N = 5.

**Figure 2:**
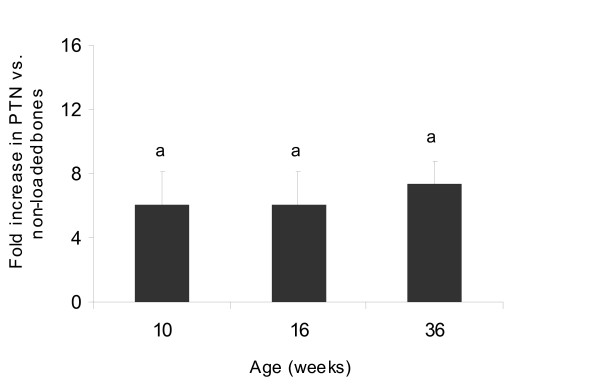
**Expression levels of PTN gene as a function of age**. The y-axis represents fold increase in the PTN gene in response to 12 days of four-point bending on tibia and the x-axis represents varying age groups of female B6 mice. Values are mentioned as mean ± SD. ^a^p < 0.05 vs. non-loaded bones, N = 5.

In the previous study using whole genome microarray analysis, we reported that ML caused a significant increase in PTN expression in the good responder female B6 mouse [7]. In order to confirm this finding, we evaluated temporal changes in PTN expression during 2 weeks of four-point bending. We found that ML caused a 2-fold increase in PTN expression as early as 2 days that was sustained during the entire 2 weeks of mechanical loading (Figure 1). Furthermore, ML effects on PTN expression was seen in three different age groups of mice, 10-, 16- and 36-weeks (Figure 2). These data demonstrate that PTN is a mechanoresponsive gene in the bones of mice. In contrast to this in vivo finding, an in vitro study using cultured human osteoblast cells have shown that PTN expression decreases in response to mechanical stimulation [19]. Although we cannot fully explain this discrepancy between our data and the in vitro study, possible explanations include: 1) Osteoblast responsiveness to mechanical loading may differ in vivo vs. in vitro. 2) Type of mechanical loading and the amount of strain utilized were different between the two studies.

It is well established that the amount of mechanical strain exerted by a given load is largely dependent on the cross sectional area (moment of inertia) such that a mouse with a large cross sectional area will experience lower mechanical strain and vice versa [1,20]. In order to assure that the difference in the bone responsiveness to loading between PTN KO mice and controls is not due to difference in the mechanical strain, we measured the bone size by pQCT at tibia mid diaphysis and calculated the mechanical strain using a mathematical model (Stephen C. Cowin: Bone Mechanics Hand book, 2nd edition, 2001, chapter: Techniques from mechanics and imaging) for both sets of mice before the loading. We found that there is no significant difference in the bone size (4.55 mm vs. 4.69 mm, p = 0.50) as well as in the mechanical strain for 9 N (6310με vs.6351 με, p = 0.91) between the PTN KO mice and controls. Thus, the applied load was the same for both sets of mice.

**Figure 3:**
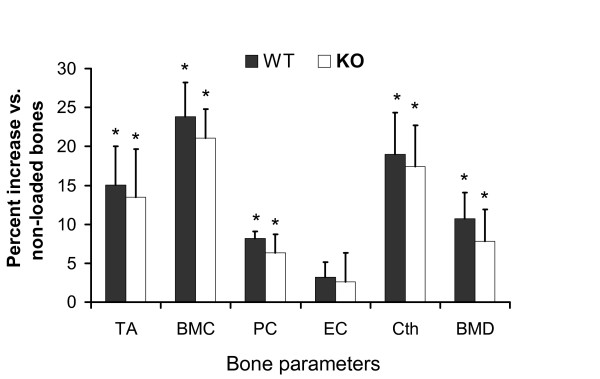
**Changes in bone parameters in response to ML on 10-wk female PTN KO and control mice**. Values are mentioned as mean ± SD. The y-axis represents percent increase in bone parameters in response to four-point bending on tibia and x-axis represent skeletal parameters. TA, Total area; BMC, bone mineral content; PC, periosteal circumference; EC, endosteal circumference; Cth, cortical thickness and BMD, bone mineral density. *p < 0.05 vs. corresponding non-externally loaded tibiae, N = 7.

To determine if ML induced increase in PTN expression contributes to anabolic effects of ML, we performed four-point bending using a load (9 N) that has been shown to exert significant changes in BMD [1]. If PTN is an important mediator of skeletal anabolic response to loading, we anticipated PTN KO mice to show reduced anabolic effects of ML on bone. We found that there was, indeed, a small reduction in ML response in the PTN KO mice (Figure 3, see additional file 1); however, these changes were not statistically significant (Since there was no difference in the response, we did not proceed any further with histomorphometeric analysis). A potential explanation for the lack of significant differences between the control and KO mice is that PTN disruption could lead to increased expression of other molecules which share similar functional properties to compensate for the loss of PTN. For example, midkine belong to the family of HB-GAM as PTN that has been shown to have similar functional properties. Mice with midkine or PTN deficiency have been reported to have normal low auditory response while mice with both gene deficits showed impaired auditory response [21]. A similar observation has been also reported with regard to fertility. Mice with disruption of both genes were infertile while mice with deficiency in either midkine or PTN gene were able to produce similar number of offspring's [22]. Another study has shown that mouse with absence of PTN gene resulted in normal skeletal growth and this is likely due to an increase in midkine expression as evident from their microarray data [23]. Overall, these findings suggest that factors of the same family are exhibiting overlapping function and thus, interfering with the activity of one factor may not necessarily lead to disruption of physiological activities such as bone formation response to loading. The issue of whether disruption of both PTN and midkine will exert a greater deficit in the skeletal anabolic response to ML compared to individual knock out requires further study.

## Competing interests

The authors declare that they have no competing interests.

## Authors' contributions

All experimental procedures, data analysis and study coordination were carried out by CK. SM contributed to the design, data interpretation and manuscript preparation. All authors read and approved the final manuscript.

## Supplementary Material

Additional file 1 **pQCT measurement of bone parameters.** The data in this file shows absolute changes in bone parameters in response to loading between PTNKO and control mice. Click here for file
