# Supplementary material for: Lack of anabolic response to skeletal loading in mice with targeted disruption of the pleiotrophin gene
Source: BMC Res Notes. 2008 Dec 1;1:124. doi: 10.1186/1756-0500-1-124 (PMC2612677; doi:10.1186/1756-0500-1-124)
Supplement: Additional file 1 — pQCT measurement of bone parameters. The data in this file shows absolute changes in bone parameters in response to loading between PTNKO and control mice. [file 1756-0500-1-124-S1.doc]

Table-1

| Bone parameters | PTN KO | | WT | |
| --- | --- | --- | --- | --- |
|  | Mean ± SD | | Mean ± SD | |
|  | Non-Loaded | Loaded | Non-Loaded | Loaded |
| Bone mineral content (mg) | 1.16 ± 0.10 | 1.40 ± 0.07* | 1.17 ± 0.14 | 1.44 ± 0.13* |
| Periosteal circumference (mm) | 4.55 ± 0.23 | 4.83 ± 0.23* | 4.69 ± 0.21 | 5.03 ± 0.26* |
| Endosteal circumference (mm) | 3.36 ± 0.18 | 3.44 ± 0.20 | 3.53 ± 0.17 | 3.69 ± 0.24 |
| Total vBMD (mg/cm^3^) | 881 ± 52 | 949 ± 55* | 839 ± 40 | 926 ± 27* |
| Cortical thickness (mm) | 0.27 ± 0.01 | 0.31 ± 0.01* | 0.25 ± 0.01 | 0.29 ± 0.01* |

*p<0.05 vs. corresponding non-externally loaded tibiae, N=7
